# Representing mutations for predicting cancer drug response

**DOI:** 10.1093/bioinformatics/btae209

**Published:** 2024-06-28

**Authors:** Patrick Wall, Trey Ideker

**Affiliations:** Department of Bioengineering, University of California San Diego, La Jolla, CA 92093, United States; Department of Bioengineering, University of California San Diego, La Jolla, CA 92093, United States; Department of Medicine, University of California San Diego, La Jolla, CA 92093, United States; Department of Computer Science and Engineering, University of California San Diego, La Jolla, CA 92093, United States

## Abstract

**Motivation:**

Predicting cancer drug response requires a comprehensive assessment of many mutations present across a tumor genome. While current drug response models generally use a binary mutated/unmutated indicator for each gene, not all mutations in a gene are equivalent.

**Results:**

Here, we construct and evaluate a series of predictive models based on leading methods for quantitative mutation scoring. Such methods include VEST4 and CADD, which score the impact of a mutation on gene function, and CHASMplus, which scores the likelihood a mutation drives cancer. The resulting predictive models capture cellular responses to dabrafenib, which targets BRAF-V600 mutations, whereas models based on binary mutation status do not. Performance improvements generalize to other drugs, extending genetic indications for PIK3CA, ERBB2, EGFR, PARP1, and ABL1 inhibitors. Introducing quantitative mutation features in drug response models increases performance and mechanistic understanding.

**Availability and implementation:**

Code and example datasets are available at https://github.com/pgwall/qms.

## 1 Introduction

A basic mode of precision oncology is to scan the tumor genome for genetic alterations, typically activating mutations in oncogenes, that can be specifically recognized by targeted inhibitors. For example, dabrafenib competes with ATP for binding to the BRAF catalytic site and is thus indicated for BRAF V600+ melanoma (Maloney *et al.* 2021). Similarly, EGFR L858R mutations activate EGFR signaling, indicating targeted inhibitors like osimertinib (Gijtenbeek *et al.* 2023); PIK3CA mutations indicate the use of inhibitors like alpelisib (André *et al.* 2019); and so on. While such targeted therapeutics have been transformative, a substantial proportion of patients fail to respond despite having the supposed biomarkers of a successful response (Hu and Dignam 2019). This challenge of distinguishing responders from non-responders extends to non-targeted chemotherapeutics, where a precise set of molecular indications is often lacking.

**Figure 1.**
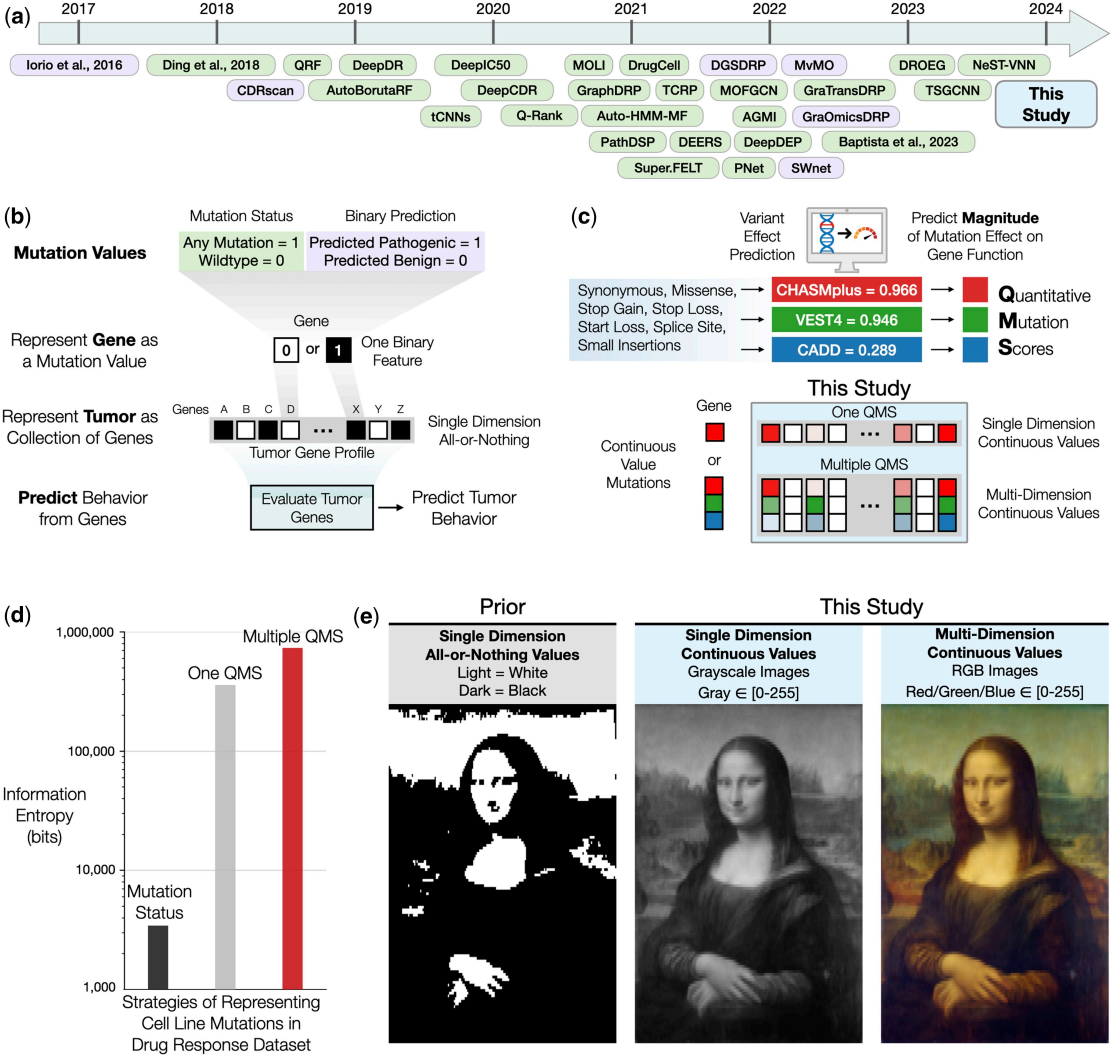
From all-or-nothing representations to scored mutations. (a) Previous models for drug response prediction (see also Supplementary Table S1) arranged beneath time of publication. Strategies for representing mutations organized by text color corresponding to those in panel (b). (b) Previous strategies for representing somatically mutated genes as an all-or-nothing mutation status indicator (mutated = 1, not mutated = 0), which treats all mutations as equal. Genes values are integrated to create a molecular gene profile of a tumor used in drug response prediction models. (c) Three variant prediction algorithms (CHASMplus, VEST4, CADD) are used to generate mutation-specific quantitative mutation scores (QMS) values for a variety of mutation types. QMS are continuous values [0,1) that predict how likely a particular mutation is to alter the function of a gene. In QMS profiles, mutated genes are represented by the QMS value corresponding to their mutation (One QMS), or as the set of QMS values from multiple QMS algorithms (Multiple QMS), with color scales representing larger (bright colors) or smaller (faded colors) values. (d) The information entropy (*y*-axis; Shannon entropy, log2 bits) of three strategies to represent mutations present in a drug response dataset (*x*-axis): Mutations represented as all-or-nothing gene mutation status (left, black); Mutations represented by continuous values of one QMS algorithm (middle, gray); Mutations represented by continuous values of three QMS algorithms (right, red). (e) Image of the Mona Lisa displayed as one-dimensional binary values (black and white, left), one-dimensional continuous values (grayscale, middle), or three-dimensional continuous values (RGB color, right).

Beyond activating mutations that are directly targeted, many predictive models have been recently introduced that integrate genetic alteration information across many, if not all, human genes (Fig. 1a; Supplementary Table S1) (Partin *et al.* 2023). The rationale for these expanded models is that genetic modulators of drug response can occur not only in the targeted protein, but also in proteins that physically or functionally interact with the target in the same or related molecular pathway. In expanding to mutational states of many genes, these models have generally not attempted to resolve which individual nucleotides or amino acids are affected in each gene, or their functional effects. Rather, each gene is assigned either a 0 or 1 based on the absence or presence of non-synonymous coding mutations (Fig. 1b). Combining mutation values across genes creates a genomic profile of a tumor, which multi-gene models use to predict tumor behaviors. Examples of approaches using this type of binary encoding include GraphDRP, DrugCell, and PNet, among others (Fig. 1a, all models listed in Supplementary Table S1). Some of these models pre-select particular types of mutations (e.g. DeepDEP, DeepDR: presence/absence of any SNV/indel/splice site/nonsense) or those with prior functional associations, such as mutations to kinases only (DEERS), mutations already correlated with drug responses (QRF), or genes with high mutation frequency in cancer cell lines (DGSDRP).

While these approaches have shown promising successes, it remains that some gene mutations clearly impact a drug response more than others, and to varying degrees. The question then is how to generally assess the effects of mutations observed across a large set of genes/proteins, not just for the single protein specifically targeted by a drug. Relevant to this task is the growing collection of variant effect prediction algorithms, which estimate the likelihood of a genetic variant impacting its protein function (Horne and Shukla 2022) (Fig. 1c). These algorithms have not yet been widely used in drug response models, however, and when they have been it is only as a pre-filter to remove mutations with low expected effect (Koras *et al.* 2020).

Here, we evaluate the benefit of integrating variant effect prediction algorithms directly in drug response models. In the assessment that follows, we find that associating somatic mutations with predicted impact scores, which we refer to as quantitative mutation scoring (QMS), not only increases the information of somatic mutation features (Fig. 1d) but enhances model accuracy and mechanistic interpretability. Moreover, multiple mutation scores can be combined to create a multi-dimensional representation of gene mutations, providing richer and more expressive information than is captured by any single QMS method. By analogy, if binary gene mutation states (0/1) are akin to a black-and-white photograph (Fig. 1e, left), moving to QMS reveals a more nuanced grayscale image (Fig. 1e, middle), while combining continuous values across multiple dimensions yields a full-color picture (Fig. 1e, right).

## 2 Materials and methods

### 2.1 Tumor cell line datasets

Datasets were compiled from GDSC (Yang *et al.* 2012) and CTRP (Rees *et al.* 2016), resulting in response data for 1,244 cell lines and 26 anti-cancer agents. Drug response was reported as area under dose–response curve (AUDRC, continuous values, 0 = total cell death, 1 = no effect, >1 = cell growth). Repeated drug/cell tests were averaged. Cell line somatic mutations were accessed from the Cancer Cell Line Encyclopedia (CCLE) DepMap portal (23Q1 release). A set of 702 genes was constructed from the union of genes contained within the FoundationOne CDx (Frampton *et al.* 2013), Tempus xT (Beaubier *et al.* 2019), Project GENIE (Smyth *et al.* 2020), and PALOMA Trial (Lira *et al.* 2017) gene panels. Filtering by our panel genes and drug cell lines resulted in 61,284 somatic mutations used in this study.

### 2.2 Scoring mutations and gene features

Variant effect prediction algorithms were selected by their superior performance in five cancer benchmark tasks, as examined in a previous study (Chen *et al.* 2020): (i) identifying pathogenic mutations clustered in 3D; (ii) identifying known cancer driver mutations; (iii) identifying mutations impacting TP53 kinase activity; (iv) identifying mutations driving *in vitro* cell growth; (v) identifying tumor-forming mutations in patient-derived xenograft models. From these data, we selected three high-performing algorithms that generate continuous value scores for somatic mutations: CHASMplus and VEST4 predict a continuous value ∈ [0, 1) denoting the probability a variant drives cancer or impacts protein function, respectively. CADD generates a value ∈ [0, 99] denoting the likelihood a mutation is deleterious to protein function, which was normalized ∈ [0, 0.99). Mutation scores were generated with OpenCRAVAT (Pagel *et al.* 2020), an open-source variant annotation platform. Algorithms generated at least one score for 54,757 mutations (89.3% of somatic mutations). Tumor cell lines were represented as a collection of gene states, wherein each gene was described by a single value. Binary mutation features assigned unmutated genes a 0 and genes with one or more somatic mutations a 1. QMS features assigned unmutated genes a 0 and genes with a somatic mutation the QMS value of its mutation (maximum QMS value if multiple mutations in a gene). A binary “not scored” feature was created for mutations not scored by any of the three QMS algorithms, which was concatenated to QMS input features during training.

### 2.3 Drug panel selection

Clinical indicators of drug responses were collected from OncoKB, a precision oncology database identifying drugs sensitive/resistant to specific genetic alterations based on varying levels of evidence. From these data, we identified a panel of 23 current cancer drugs having FDA-approved somatic mutation biomarkers. Three additional non-targeted chemotherapeutic agents were included in our panel based on the availability of relevant clinical genomics datasets.

### 2.4 Neural network architecture and training

For each drug, we created models to compute the AUDRC of a tumor from the somatic mutation features of its genes. For tumor *t*, *F*($X_{t}$) = ${\bar{y}}_{t}$, where *F* is the drug response model and $\bar{y}$ is the predicted drug response (i.e. AUDRC). $X_{t}$ is a matrix of gene features of size (702 genes × number of gene features) (binary mutations: 1 feature; 1, 2, or 3-QMS configurations: 2, 3, or 4 features, respectively; i.e. QMS features + the “not scored” feature). Each model was a feed-forward neural network with six layers, 1–6. Starting from layer 1, each layer contained 702*x, 512, 512, 2048, 36, and 4 neurons, respectively, where x is the number of input features. Layer 1 neurons were partitioned by gene, such that each of the 702-panel genes was allocated x neurons. The activation states **h** of these gene embeddings were concatenated and passed forward to the next layer. The multidimensional activation state **h** for layer “a” was calculated by the transfer function *T*($x_{a}$) = Batchnorm(tanh(Linear(Dropout($x_{a}$)))) = $h_{a}$.

*T* maps the inputs $x_{a}\in R^{c}\to R^{d}$, where *c* is the number of input features and *d* is the number of layer neurons. Linear is a linear transformation parameterized by $W^{T}x_{a}\;+\;b_{a}$, with weight matrix $W\in R^{c,d}$ and bias vector b. Dropout is the dropout function, Batchnorm is the batch normalization function, and tanh is the hyperbolic tangent activation function. We applied a linear transformation to the final layer (with four neurons) to predict $\bar{y}$.

Models were trained to minimize the mean squared error (MSE) of the real versus predicted AUDRC values (*y* versus $\bar{y}$, respectively), using the objective function: *O* = MSE(*y*,$\bar{y}$) + $\lambda ∥W{∥}_{2}$. The term on the right is the $\ell_{2}$ norm penalty for network weights *W* parameterized by weight decay parameter *λ*. Models were trained using mini-batch stochastic gradient descent with batch size = 32 and the AdamW optimizer. We used the Pytorch adjustable learning rate (torch.optim.ReduceLROnPlateau, starting at learning rate of 0.014) to reduce the learning rate by 80% every 10 epochs without validation set loss improvement. An early stop method terminated training if validation loss did not improve by at least *ϵ* = 1 × 10^–4^ after 20 epochs.

### 2.5 Control for overfitting

Model overfitting was controlled and assessed using several key measures. First, the model training procedure was built to incorporate multiple regularization methods, including early stop, dropout, and weight decay functions (see previous section). Second, model performance was assessed by nested cross validation, a conservative technique often used in machine learning to reliably evaluate the performance and generalizability of a model, especially when the dataset size is limited (Parvandeh *et al.* 2020). By apportioning samples (cell lines) into train/validate/test partitions, model optimization (fitting of parameters and hyperparameters) was fully insulated from the final performance assessment, which was conducted for tumor cell lines not seen previously during any earlier stage of model training or validation. Third, we observed that random shuffling of drug responses across cell lines broke model performance almost entirely (Supplementary Fig. S1), suggesting the models are not overfit. Finally, model generalizability was evaluated by the degree of transferability from cell lines to patient tumor biopsies, as well as the transferability from drugs used during training to alternate drugs against the same targets (Fig. 4, Supplementary Fig. S2).

### 2.6 Computational complexity of model training

QMS models encode gene features with a square matrix *O*(|features${|}^{2}$). Roughly, the training complexity is [*O*(|features${|}^{2}$*|genes|) + *O*(|genes|*n_1_) + $\Sigma_{n\neq 1}$*O*(*n*${\;}_{i-1}$*n_i_)] * epochs, where *i* is the model layer and *n_i_* are the number of neurons of that layer. Models never exceed four input features (CHASMplus, VEST4, CADD, and the “not scored” binary mutation features), so complexity is dominated by the deeper model layers. Each (1244 cell lines × 702 genes) PyTorch float tensor requires approximately 5 MB of memory. Concatenating multiple QMS arrays into a single tensor object further reduces the memory requirements per extra QMS feature. Additional computational details are provided (Supplementary Table S2). The time complexity of model training is not significantly affected by multi-QMS configurations.

### 2.7 Alternate models for predicting drug responses

QMS model predictions were compared against DrugCell (Kuenzi *et al.* 2020) and DeepCDR (Liu *et al.* 2020), two previously published methods developed to predict responses of many tumor cell line drug pairs. These models were retrained by 5-fold cross-validation on the same cell line drug response dataset used to train QMS models, which ensures all models have observed the drugs of our drug panel during training. Test sets consisted of held-out cell lines.

### 2.8 Interpretation and importance scores

Models were interpreted by gradient analysis. Gradients quantify the influence of a model feature on the final prediction; measuring the size of the gradient can be considered as the importance of the feature. For any model feature f in our network, we defined the gradient as the change in model prediction $\bar{y}$ with respect to the feature f. Thus, *G_f_* = $\partial \bar{y}/\partial f$ and was calculated via chain rule and accessed using the torch.Tensor.register_hook() method. The importance of each model feature was calculated by the $\ell_{2}$ norm of the feature gradients. Importance scores were averaged across nested cross validation models (test set predictions; unseen tumors).

### 2.9 Predicting sensitivity to BRAF inhibitors in cutaneous melanoma

**Figure 2.**
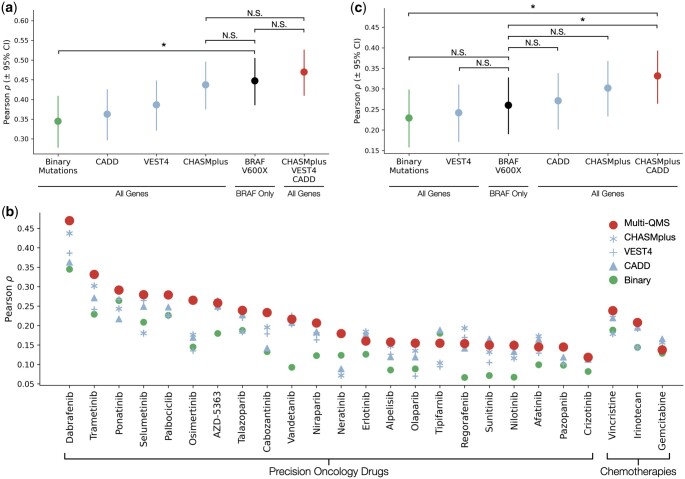
Evaluation of alternative feature sets in predicting tumor drug responses. (**a**) Pearson correlation of model predictions versus measured responses to dabrafenib (*y*-axis), with six alternative feature sets (*x*-axis) ordered by increasing predictive performance. Feature sets from left-to-right include binary mutation status of clinical panel genes (unmutated = 0; any mutation in coding sequence = 1); continuous mutation scores of these genes using each of the three mutation scoring algorithms (CADD, VEST4, CHASMplus); binary mutation status of the BRAF 600 amino-acid residue only (V = 0; other = 1); and a combination of all three scored features. Test set predictions are from 5-fold nested cross validation runs with 70%/15%/15% (train/validation/test) splits. Error bars show 95% confidence intervals. *P < .05 by Fisher’s *r*-to-*z* transform. NS = not significant. (**b**) As for (a), showing average Pearson correlation of model predictions versus actual drug responses (y-axis) across a panel of 23 targeted precision oncology inhibitors and three chemotherapies (x-axis) using various feature set configurations (larger red circle = multi-QMS model, blue asterisk = CHASMplus, blue plus sign = VEST4, blue triangle = CADD, smaller green circle = binary mutations). (**c**) As for (a), but predicting cell line responses to trametinib, a MEK inhibitor also indicated for use by the presence of BRAF V600X mutations.

A clinical genomics dataset relevant to a cutaneous melanoma cohort (Van Allen *et al.* 2014) was accessed from cBioPortal (Cerami *et al.* 2012) (39 patients; largest publicly available BRAF inhibitor dataset). Patients were treated with either dabrafenib or vemurafenib (35/39 BRAF V600E/K${\;}^{+}$, 28/39 treated with vemurafenib, 11/39 treated with dabrafenib), and outcomes were recorded as RECIST classes: Partial response (PR); Stable disease (SD); Progressive disease (PD). Patients exhibiting partial response were considered sensitive to BRAF inhibition, and patients exhibiting stable/progressive disease were considered resistant. Because the drugs have similar molecular mechanisms (Haling *et al.* 2014), all samples were used to predict responses regardless of BRAF inhibitor. Somatic mutations were limited to those present in our gene panel and were evaluated by QMS methods (CHASMplus, VEST4, CADD). Somatic mutation profiles were constructed for each QMS model as well as for the binary mutation models. Models previously trained on dabrafenib cell-line data were used to predict drug responses (Fig. 2a; multi-QMS and binary models). Responses and importance scores were averaged from the five nested cross validation models for each input configuration. Prediction performance was assessed by F1 score and precision–recall statistics. As model predictions were AUDRC values, patients were labeled as responsive if the predicted values were in the bottom 20th percentile of AUDRC over tumor cell lines. Otherwise, patients were labeled as non-responsive.

**Figure 3.**
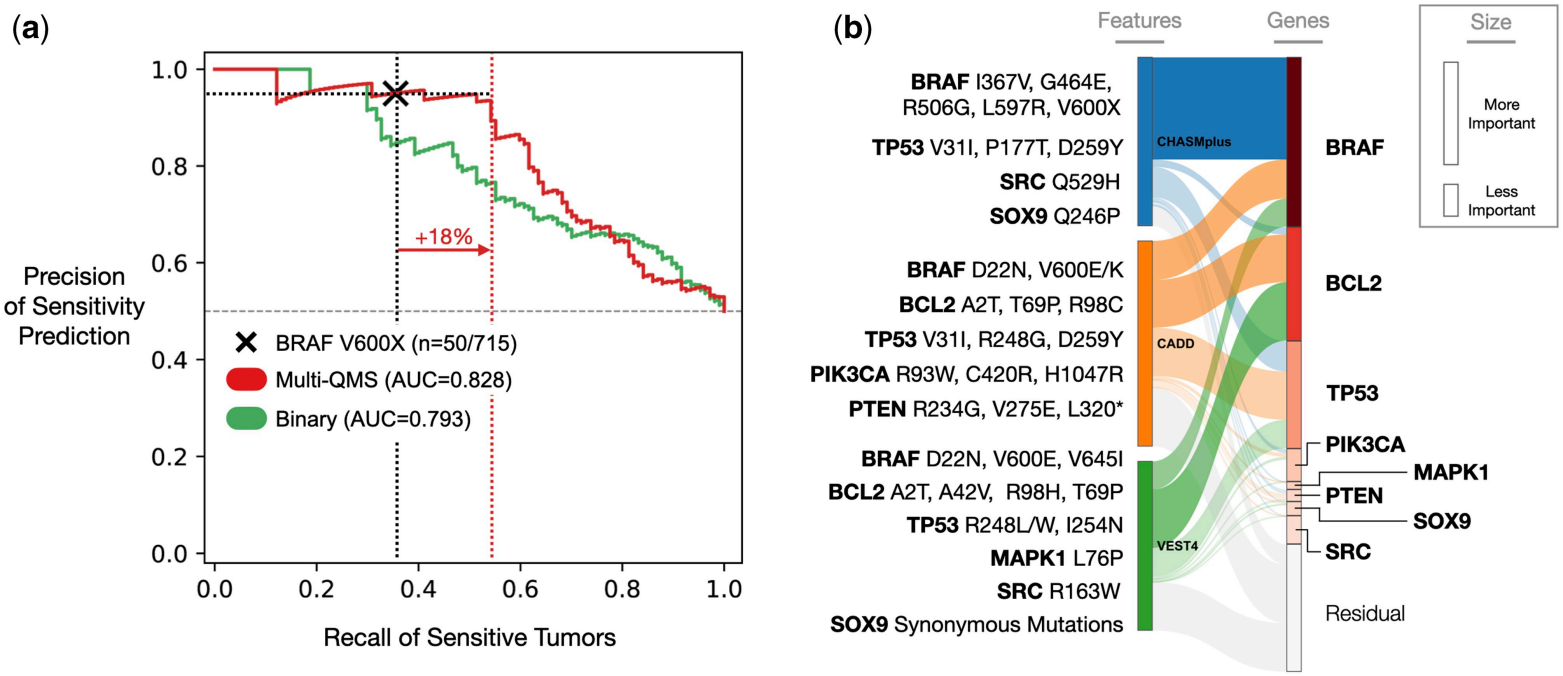
Scored mutations capture and extend gene alterations predictive of dabrafenib sensitivity. (a) Precision–recall curves as an assessment of model performance in identifying unseen tumor genotypes sensitive to dabrafenib, highlighting two feature sets: binary mutations (green) and multi-QMS scored mutations (red). Area under precision–recall curve in parentheses. Precision/recall of BRAF V600X biomarker denoted by (mutation present in 50 out of 715 test set cell lines). Recall values of BRAF V600X (vertical black dashed line) and the multi-QMS model (vertical red dashed line) are indicated, thresholded at the same 95% precision value (horizontal black dashed lines). (b) Sankey diagram illustrating how groups of QMS features (left: CHASMplus, VEST4, CADD) affect genes (right) embedded in a dabrafenib neural network model. Groups of features within each layer are represented by vertical rectangles, with height reflective of importance in model predictions. The thickness of the band connecting features denotes influence in model predictions (Section 2). Important but less influential genes are binned together and represented as the residual box (gray). Mutations identified by the model as predictive of dabrafenib response are shown (far left) next to the QMS method the model used to identify the mutation (mutations are repeated if the mutation was recognized by multiple QMS methods). Image generated by Plotly (version 5.13).

### 2.10 Predicting sensitivity to EGFR inhibitors in lung cancer

A clinical genomics dataset relevant to non-small cell lung cancer (Choudhury *et al.* 2023) was accessed from cBioPortal (Cerami *et al.* 2012). Data were extracted for patients treated with osimertinib (*n* = 215) and included patient outcomes (overall survival, months) and exome somatic mutations from pre-treatment tumor biopsies. Somatic mutations were limited to those present in our gene panel, and mutation profiles were constructed for each QMS model (CHASMplus, VEST4, CADD, and a binary “not scored” feature set) as well as for binary mutation models. Models trained on cell-line osimertinib data (Fig. 2c) were used to predict patient responses. Patients were labeled as responsive or non-responsive as for cutaneous melanoma (above section). Stratification performance was assessed by hazard ratio and C-index (calculated by the Python library “Lifelines”).

## 3 Results

### 3.1 Tumor cell drug responses and mutation scoring

We accessed drug treatment data for 26 chemical agents measured across each of 1,244 tumor cell lines (Section 2). Each tumor cell drug response was summarized as the AUDRC. Exome-wide somatic mutations were accessed for each cell line, focusing on alterations in 702 genes commonly screened in clinical gene panels (Section 2). These somatic mutations were scored by three leading QMS algorithms: CHASMplus, a cancer-specific algorithm that assigns the likelihood a mutation drives cancer (Tokheim and Karchin 2019); VEST4, an algorithm predicting the likelihood a mutation alters protein function (Carter *et al.* 2013); and CADD, a complementary algorithm predicting altered protein function (Rentzsch *et al.* 2021). Given this information, cell lines were represented as a profile of gene mutation states, with each gene represented by the maximum QMS score of its (possibly multiple) somatic mutations. Scores from each algorithm were either treated separately, resulting in one score per gene (single-QMS), or using scores for multiple algorithms concatenated together (multi-QMS). For comparative benchmarking, a third representation was constructed in which QMS values were replaced by binary (0/1) mutation indicators for each gene (binary values).

### 3.2 Improved accuracy of QMS models over binary gene mutations

An instructive test case for QMS prediction is the response of tumor cells to dabrafenib, a small molecule designed to specifically target V600X activating mutations in the BRAF kinase (X denotes any amino acid change). BRAF V600X mutations specifically elicit dabrafenib sensitivity, whereas other BRAF mutations generally do not (Maloney *et al.* 2021). Notably, all three QMS algorithms scored BRAF V600X mutations as particularly deleterious (CHASMplus = 0.996, VEST4 = 0.946, and CADD = 0.289 representing 99th, 91st, and 87th percentiles of all mutation scores across cell lines). A key question then was whether drug response models based on these QMS features would recognize the V600X alteration as informative for prediction, or if these models would instead give preferential attention to mutation scores for the many other genes provided as input.

Accordingly, we constructed a series of deep neural network models that use gene mutation features (single-QMS, multi-QMS, or binary values; see previous section) to predict the response of each tumor cell line to dabrafenib treatment (Fig. 2a). To guard against data leakage and overfitting, models were trained and tested using a rigorous nested 5-fold cross validation procedure, in which the collection of cell lines is partitioned into 70%/15%/15% splits for training, validation, and test phases (Section 2). Performance was estimated on the held-out lines using Pearson correlation. Analyzing these results, we found that use of the single CHASMplus feature for each gene significantly outperformed use of binary mutations (Fig. 2a) and yielded essentially the same performance as directly encoding knowledge of the BRAF V600X indicator. Combining multiple mutation scores (multi-QMS model) showed a further increase in performance above all other models, although this effect was not significant.

We further expanded our assessment to models constructed for each of 26 precision oncology therapies (Section 2). We found that at least one of the single-QMS models outperformed binary mutations for all 26 drugs (Fig. 2b). Multi-QMS configurations outperformed the binary model in 25/26 cases, and they usually, but not always, outperformed single-QMS configurations (20/26 drugs). One example of this was trametinib, a selective inhibitor of MEK1 downstream of BRAF. In this case, single QMS models performed nearly equivalently to knowledge of BRAF V600X (Fig. 2c), as did binary mutations. However, combining multiple QMS significantly improved performance over all other models (Fig. 2c).

### 3.3 QMS models extend canonical biomarkers with additional mutations

To provide further insight into the dabrafenib drug response models, we next benchmarked their various feature sets against the BRAF V600X marker using precision–recall statistics. For this purpose, the collection of tumor cell lines was equally divided into dabrafenib-sensitive versus resistant classes, depending on whether the AUDRC was in the top 20% of most sensitive or resistant responses (Section 2). In this configuration, we saw that the BRAF V600X marker was very precise in predicting dabrafenib sensitivity (precision = 95%) with moderate recall of these sensitive samples (recall = 35%; Fig. 3a). We then examined the quantitative output of the multi-QMS model, which we also thresholded to sort tumor cell lines into predicted sensitive versus resistant classes; varying this sensitivity threshold traced a precision–recall curve (Fig. 3a, Section 2). From this curve, we noted that the multi-QMS model was able to maintain the precision of the BRAF V600X marker (95%) while substantially extending the recall of sensitive samples (from 35% to 53%). These results implied that QMS not only captures the effects of BRAF V600X but also other mutation features predictive of dabrafenib sensitivity.

To identify these other important features, we interpreted the multi-QMS dabrafenib neural network using a gradient-based methodology (Section 2) in combination with Sankey diagrams, which help visualize the flow of information through a network (Fig. 3b). QMS features of the BRAF gene ranked as the most important, primarily driven by BRAF CHASMplus and BRAF CADD scores. The second-most important mutated gene was BCL2, an anti-apoptosis factor that facilitates cell death during BRAF inhibition (Sullivan *et al.* 2018). Other top mutated genes TP53 (Wang *et al.* 2023), PIK3CA (Candido *et al.* 2022), MAPK1 (Long *et al.* 2014), and more, are known modulators of BRAF activity and dabrafenib response, many of which are under clinical investigation for potential adjuvant targeting strategies in combination with BRAF/MEK inhibition. While these factors had each been recognized in previous (mostly separate) studies, they had yet to be integrated within a single precision oncology model to yield accurate drug response predictions.

### 3.4 QMS models generalize to patient cohorts

**Figure 4.**
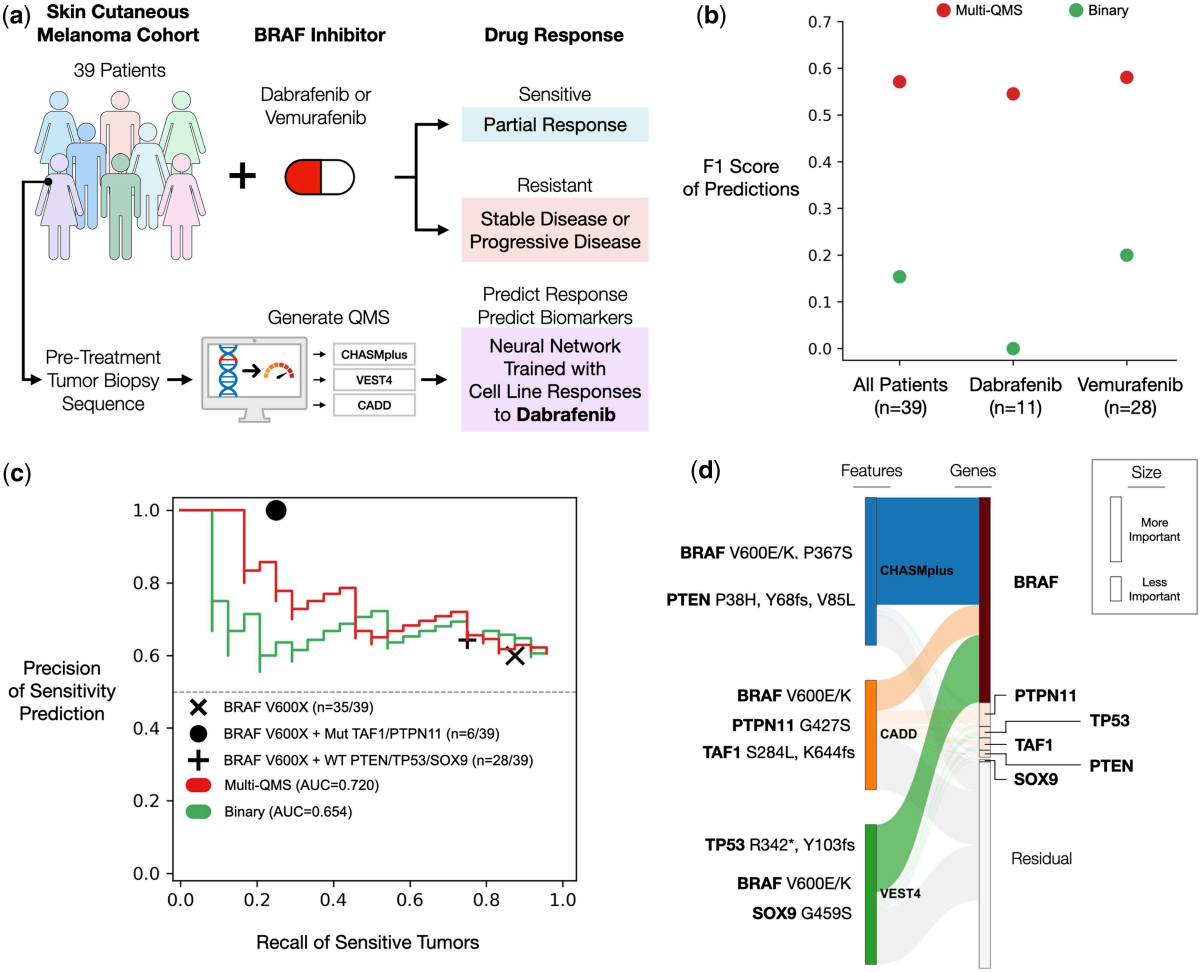
Stratification of clinical cohort and genetic markers that affect dabrafenib sensitivity or resistance. (a) Tumor biopsies of skin cutaneous melanoma (*n* = 39 patients; 28 treated with vemurafenib, 11 treated with dabrafenib) were sequenced prior to monotherapy with a BRAF inhibitor (vemurafenib or dabrafenib). Patient outcomes were recorded according to RECIST criteria. Tumor somatic mutations were used to predict drug responses in models trained from cell line data (same dabrafenib models highlighted in Fig. 2a). (b) Performance of BRAF inhibitor response prediction by using *F*1 score (reports the average of precision and recall), assessed on all patients (left), patients who received dabrafenib (middle), or patients who received vemurafenib (right). (c) Performance of BRAF inhibitor response prediction using precision–recall curves. Similar to Fig. 3a, with additional genetic markers indicating sensitivity to BRAF inhibition in the melanoma cohort. Solid black circle: BRAF V600X mutations with mutations in TAF1 or PTPN11. Plus sign: BRAF V600X mutations with wildtype PTEN, TP53, and SOX9. (d) Similar to Fig. 3b, showing the flow of genetic information between input features and genes embedded in the dabrafenib model.

Finally, we evaluated how well QMS models translate from tumor cell lines to patients. First, we examined a clinical study of 39 cutaneous melanoma patients treated with either dabrafenib or vemurafenib, another targeted inhibitor of oncogenic BRAF V600X mutations (Van Allen *et al.* 2014) (Fig. 4a, Section 2). Treatment outcomes had been recorded using the RECIST classes of partial response (PR, reduction in tumor volume), stable disease (SD, no change in tumor volume), or progressive disease (PD, increase in tumor volume). Patients with PR were considered sensitive to BRAF inhibition, and patients with stable or progressive disease were considered resistant. Tumors were biopsied before treatment and subjected to whole-exome sequencing to call somatic mutations.

Previously constructed dabrafenib predictive models (Fig. 2a) were benchmarked against patient outcomes by F1 score (Fig. 4b, Section 2) and precision–recall statistics (Fig. 4c, Section 2). Multi-QMS mutations were more predictive of patient responses than binary mutations, as we had observed earlier for tumor cell lines (Fig. 2b). Notably, dabrafenib QMS models extended to predicting outcomes of patients treated with vemurafenib (Fig. 4b). In contrast to cell lines, the BRAF V600X marker had very low precision as it was found in nearly all (35/39) patients. These results implied that the better performance of the QMS models was due to other features. In particular, gradient-based interpretation of the QMS models showed that, beyond BRAF, high importance was assigned to TP53, PTEN, and SOX9, similar to our previous findings in cell lines (Fig. 3b), as well as to TAF1 and PTPN11, which have been implicated in dabrafenib sensitivity (Wang *et al.* 2014, Harigai *et al.* 2022). Thus, the QMS model generalized to predictions of patient outcomes, as well as to drugs with similar molecular mechanisms.

Generalizability was further assessed on a cohort of non-small cell lung cancer patients who received the EGFR inhibitor osimertinib (215 patients; Supplementary Fig. S2a). Exome sequences of tumor biopsies were obtained prior to treatment, and outcomes were recorded as overall survival (months; Section 2). We found that overall survival was significantly longer in patients predicted as responsive to osimertinib by cell-line QMS models than in patients predicted as non-responsive (Supplementary Fig. S2b, Section 2; Cox proportional hazards test; *P < .05). Notably, while binary models successfully partitioned patient responses, actual patient outcomes were the inverse of what these models had predicted (Supplementary Fig. S2c). Thus, binary models recognized the importance of mutations but could not decipher their effects, whereas QMS models identified features useful for predicting sensitivity/resistance (*C*-index scores 0.56 versus 0.42, QMS versus binary).

## 4 Discussion

Here we have explored the utility of QMS, a class of approaches for representing genetic mutations in drug response models. Contrasting with previous models, which generally do not differentiate individual mutations, we evaluated each of three variant effect prediction algorithms: CADD and VEST4, which assess the likelihood of a mutation altering normal gene function, and CHASMplus, which evaluates the potential to drive cancer. First, we evaluated whether these QMS approaches distinguish mutations known to affect drug response, using the test case of dabrafenib—a targeted BRAF inhibitor that disproportionately kills tumors with BRAF V600X substitutions. QMS captured the effects of BRAF V600X mutations on dabrafenib sensitivity, whereas binary (yes/no) mutations did not. Expanding the analysis to evaluate models for 25 additional precision oncology drugs and chemotherapies revealed that QMS consistently outperformed binary mutation representations across drug responses. These models used QMS values to identify functionally significant mutations, pinpoint genes critical to drug response mechanisms, and uncover genetic indicators of drug responses beyond established biomarkers. QMS models generalized from tumor cell lines to melanoma patients treated with BRAF inhibitors, where these models not only outperformed binary mutations but also identified key molecular factors influencing BRAF inhibition.

In some cases, one QMS method was enough to capture functional variants, such as CHASMplus alone being sufficient to highlight BRAF V600X mutations (Figs 2a and 3b). In other cases, combining scores from multiple algorithms allowed models to find predictive features not available in single-QMS configurations (e.g. multi-QMS trametinib and dabrafenib models; Figs 2a, 2c and 3b). On the other hand, algorithms that generate redundant annotations can inflate computational requirements and increase the likelihood of overfitting. Ideally, multi-QMS configurations should capture orthogonal, complementary, and biologically relevant information.

The models explored here implement a two-phase approach: Individual amino acid changes are scored in a first phase, after which these scores are provided to a second modeling phase to predict response. One wonders whether better performance might be achieved by a model that directly translates amino acid changes into a prediction of tumor drug response. While such an approach should be investigated, tumors exhibit an enormous number of rare mutations; inferring the effects of each on drug responses may require a prohibitive number of observations. A two-phase approach, using QMS as an intermediate interpreter of amino acid changes, may help distinguish mutations with less training data. On the other hand, QMS does not consider drug-related effects and thus may not sufficiently resolve certain impactful mutations. Choosing a one versus two-phase approach will also depend on computational resources. During training, QMS algorithms evaluate dozens of atomic, molecular, and biological features for each mutation (CHASMplus = 95 features, VEST4 = 86 features, CADD = 63 features). Two-stage models benefit from scores that reflect these features, without including the features themselves during training.

While QMS features generally increased model performance, for some drugs, additional biological mechanisms likely influence drug responses in ways not captured by either binary or QMS mutations (Fig. 2b). For example, our models did not consider the effects of genes with multiple mutations, which might be evaluated by assigning genes with multiple mutations a 1 (and 0 otherwise), or simply representing a gene with the number of its mutations. Indeed, other binary strategies are certainly possible, such as assigning genes a 1 for mutations that score above/below some threshold. These models also do not consider structural variants, which significantly impact drugs like ponatinib (also indicated by BCR-ABL1 fusions) (Luciano *et al.* 2020). Expanding the gene set to include additional classes of molecular biomarkers is of immediate interest moving forward.

Patient outcomes are recorded with categorical (e.g. RECIST classes) and continuous value (e.g. overall survival) criteria, but drug response models are typically trained on large pre-clinical cancer drug screens that measure a killing effect (e.g. AUDRC). Tumor killing may not extend to longitudinal measures of sustained patient responses. Models aiming for translational applications might consider reconciling model predictions with relevant clinical outcomes. This analysis attempted to convert the AUDRC values predicted for patients into a label denoting the patient was likely/unlikely to respond to therapy. These cutoffs succeeded at stratifying patients, but additional readouts describing how confident a model is in its decision would be very useful.

Transitioning from binary to scored mutations not only resolved BRAF V600X from other BRAF mutations, but found additional mutations highly predictive of dabrafenib responses in both cell lines and melanoma patients (Figs 3a, 3b, 4b, 4c). One additional notable marker of sensitivity was mutations of BCL2, a regulator of responses to drugs targeting MEK/ERK and PARP pathways in multiple tissue subtypes (Valentini *et al.* 2023). BCL2 inhibitors increase sensitivity to BRAF/MEK inhibition in tumors without BRAF V600X mutations (Mukherjee *et al.* 2020), suggesting BCL2 mutations might provide a biomarker of successful BRAF/MEK inhibition in patients lacking a BRAF V600X mutation.

In summary, we have evaluated the benefit of encoding somatic mutations by quantitative values in drug response models, without requiring these models to evaluate gene sequences directly. An individual mutation can assume many different values, making it possible to recognize particular variants by the magnitude of their effects on a gene. In this way, QMS compresses an altered gene sequence to a single continuous value, which is sufficiently expressive to capture the mutation-specific molecular state of the gene. By incorporating QMS values, cancer drug response models can integrate the molecular states of many genes, identify relevant mutations, and make better predictions.

## Supplementary Material

btae209_Supplementary_Data

## Data Availability

The data underlying this article are available at https://github.com/pgwall/qms.

## References

[btae209-B1] André F , Ciruelos E, Rubovszky G et al; SOLAR-1 Study Group. Alpelisib for PIK3CA-Mutated, hormone receptor–positive advanced breast cancer. N Engl J Med 2019;380:1929–40. 31091374 10.1056/NEJMoa1813904

[btae209-B4906653] Beaubier N, , Tell R, , Lau D et al Clinical validation of the tempus xt next-generation targeted oncology sequencing assay. Oncotarget 2019;10:2384–96. 31040929 10.18632/oncotarget.26797 PMC6481324

[btae209-B2] Candido S , Salemi R, Piccinin S et al The PIK3CA H1047R mutation confers resistance to BRAF AND MEK inhibitors in A375 melanoma cells through the cross-activation of MAPK AND PI3K–Akt pathways. Pharmaceutics 2022;14:590. 35335966 10.3390/pharmaceutics14030590 PMC8950976

[btae209-B3] Carter H , Douville C, Stenson PD et al Identifying mendelian disease genes with the variant effect scoring tool. BMC Genomics 2013;14:S3. 10.1186/1471-2164-14-S3-S3 PMC3665549 23819870

[btae209-B4] Cerami E , Gao J, Dogrusoz U et al The cBio cancer genomics portal: an open platform for exploring multidimensional cancer genomics data. Cancer Discov 2012;2:401–4. 22588877 10.1158/2159-8290.CD-12-0095 PMC3956037

[btae209-B5] Chen H , Li J, Wang Y et al Comprehensive assessment of computational algorithms in predicting cancer driver mutations. Genome Biol 2020;21:43. 32079540 10.1186/s13059-020-01954-z PMC7033911

[btae209-B6] Choudhury NJ , Lavery JA, Brown S et al; AACR GENIE BPC Core Team. The GENIE BPC NSCLC cohort: a real-world repository integrating standardized clinical and genomic data for 1,846 patients with non–small cell lung cancer. Clin Cancer Res 2023;29:3418–28. 37223888 10.1158/1078-0432.CCR-23-0580 PMC10472103

[btae209-B7] Frampton GM , Fichtenholtz A, Otto GA et al Development and validation of a clinical cancer genomic profiling test based on massively parallel DNA sequencing. Nat Biotechnol 2013;31:1023–31. 24142049 10.1038/nbt.2696 PMC5710001

[btae209-B8] Gijtenbeek RGP , Damhuis RAM, van der Wekken AJ et al Overall survival in advanced epidermal growth factor receptor mutated non-small cell lung cancer using different tyrosine kinase inhibitors in The Netherlands: a retrospective, nationwide registry study. Lancet Reg Health Eur 2023;27. 10.1016/j.lanepe.2023.100592 PMC9932646 36817181

[btae209-B9] Haling JR , Sudhamsu J, Yen I et al Structure of the BRAF-MEK complex reveals a kinase activity independent role for BRAF in MAPK signaling. Cancer Cell 2014;26:402–13. 25155755 10.1016/j.ccr.2014.07.007

[btae209-B10] Harigai R , Sato RYO, Hirose C et al Mutation of PTPN11 (Encoding SHP-2) promotes MEK activation and malignant progression in neurofibromin-deficient cells in a manner sensitive to BRAP mutation. Cancers 2022;14:2377. 35625983 10.3390/cancers14102377 PMC9140047

[btae209-B11] Horne J , Shukla D. Recent advances in machine learning variant effect prediction tools for protein engineering. Ind Eng Chem Res 2022;61:6235–45. 36051311 10.1021/acs.iecr.1c04943 PMC9432854

[btae209-B12] Hu C , Dignam JJ. Biomarker-driven oncology clinical trials: key design elements, types, features, and practical considerations. JCO Precis Oncol 2019;3. 10.1200/PO.19.00086 PMC7446374 32923854

[btae209-B13] Koras K , Juraeva D, Kreis J et al Feature selection strategies for drug sensitivity prediction. Sci Rep 2020;10:9377. 32523056 10.1038/s41598-020-65927-9 PMC7287073

[btae209-B14] Kuenzi BM , Park J, Fong SH et al Predicting drug response and synergy using a deep learning model of human cancer cells. Cancer Cell 2020;38:672–84.e6. 33096023 10.1016/j.ccell.2020.09.014 PMC7737474

[btae209-B15] Lira ME , Xie T, Deng S et al Abstract 2749: liquid biopsy testing allows highly-sensitive detection of plasma cfDNA mutations in 87 breast cancer-related genes. Cancer Res 2017;77:2749.

[btae209-B16] Liu Q , Hu Z, Jiang R et al DeepCDR: a hybrid graph convolutional network for predicting cancer drug response. Bioinformatics 2020;36:i911–8. 33381841 10.1093/bioinformatics/btaa822

[btae209-B17] Long GV , Fung C, Menzies AM et al Increased MAPK reactivation in early resistance to dabrafenib/trametinib combination therapy of BRAF-mutant metastatic melanoma. Nat Commun 2014;5:5694. 25452114 10.1038/ncomms6694

[btae209-B18] Luciano L , Annunziata M, Attolico I et al The multi-tyrosine kinase inhibitor ponatinib for chronic myeloid leukemia: real-world data. Eur J Haematol 2020;105:3–15. 32145037 10.1111/ejh.13408

[btae209-B19] Maloney RC , Zhang M, Jang H et al The mechanism of activation of monomeric B-Raf V600E. Comput Struct Biotechnol J 2021;19:3349–63. 34188782 10.1016/j.csbj.2021.06.007 PMC8215184

[btae209-B20] Mukherjee N , Amato CM, Skees J et al Simultaneously inhibiting BCL2 AND MCL1 is a therapeutic option for patients with advanced melanoma. Cancers 2020;12:2182. 32764384 10.3390/cancers12082182 PMC7464298

[btae209-B0304519] Pagel KA, , Kim R, , Moad K et al Integrated informatics analysis of cancer-related variants. JCO Clin Cancer Inform 2020;4:310–7. 32228266 10.1200/CCI.19.00132 PMC7113103

[btae209-B21] Partin A , Brettin TS, Zhu Y et al Deep learning methods for drug response prediction in cancer: predominant and emerging trends. Front Med 2023;10. 10.3389/fmed.2023.1086097 PMC9975164 36873878

[btae209-B22] Parvandeh S , Yeh H-W, Paulus MP et al Consensus features nested cross-validation. Bioinformatics 2020;36:3093–8. 31985777 10.1093/bioinformatics/btaa046 PMC7776094

[btae209-B23] Rees MG , Seashore-Ludlow B, Cheah JH et al Correlating chemical sensitivity and basal gene expression reveals mechanism of action. Nat Chem Biol 2016;12:109–16. 26656090 10.1038/nchembio.1986 PMC4718762

[btae209-B24] Rentzsch P , Schubach M, Shendure J et al CADD-Splice—improving genome-wide variant effect prediction using deep learning-derived splice scores. Genome Med 2021;13:31. 33618777 10.1186/s13073-021-00835-9 PMC7901104

[btae209-B25] Smyth LM , Zhou Q, Nguyen B et al; AACR Project GENIE Consortium. Characteristics and outcome of AKT1E17K-Mutant breast cancer defined through AACR project GENIE, a clinicogenomic registry. Cancer Discov 2020;10:526–35. 31924700 10.1158/2159-8290.CD-19-1209 PMC7125034

[btae209-B26] Sullivan RJ , Mehnert J, Tawbi H et al First in human, dose escalation trial of the combination of dabrafenib, trametinib, and navitoclax in patients with BRAF mutant solid tumors. In: *Proceedings of the AACR-NCI-EORTC International Conference, Philadephia, PA, USA. 2018*. Dublin, Ireland: Molecular Targets and Cancer Therapeutics.

[btae209-B27] Tokheim C , Karchin R. CHASMplus reveals the scope of somatic missense mutations driving human cancers. Cell Syst 2019;9:9–23.e8. 31202631 10.1016/j.cels.2019.05.005 PMC6857794

[btae209-B28] Valentini E , Di Martile M, Brignone M et al Bcl-2 family inhibitors sensitize human cancer models to therapy. Cell Death Dis 2023;14:441. 37460459 10.1038/s41419-023-05963-1 PMC10352371

[btae209-B29] Van Allen EM , Wagle N, Sucker A et al; Dermatologic Cooperative Oncology Group of Germany (DeCOG). The genetic landscape of clinical resistance to RAF inhibition in metastatic melanoma. Cancer Discov 2014;4:94–109. 24265153 10.1158/2159-8290.CD-13-0617 PMC3947264

[btae209-B30] Wang H , Curran EC, Hinds TR et al Crystal structure of a TAF1-TAF7 complex in human transcription factor IID reveals a promoter binding module. Cell Res 2014;24:1433–44. 25412659 10.1038/cr.2014.148 PMC4260347

[btae209-B31] Wang X , Xie Q, Ji Y et al Targeting KRAS-mutant stomach/colorectal tumors by disrupting the ERK2-p53 complex. Cell Rep 2023;42:111972. 36641751 10.1016/j.celrep.2022.111972

[btae209-B32] Yang W , Soares J, Greninger P et al Genomics of drug sensitivity in cancer (GDSC): a resource for therapeutic biomarker discovery in cancer cells. Nucleic Acids Res 2012;41:D955–61. 23180760 10.1093/nar/gks1111 PMC3531057

