# Supplementary material for: Representing mutations for predicting cancer drug response
Source: Bioinformatics. 2024 Jun 28;40(Suppl 1):i160–8. doi: 10.1093/bioinformatics/btae209 (PMC11256944; doi:10.1093/bioinformatics/btae209)
Supplement: btae209_Supplementary_Data [file btae209_supplementary_data.zip › btae209_Supplementary_Data/Wall.178.sup.5.docx]

| **Supplementary Table 2: Representative computational requirements for training QMS models^†^.** | | | |
| --- | --- | --- | --- |
| **# Features / Gene^‡^** | **Max Memory (MB)*** | **Train Time (m)*** | **Early Stop Tolerance (Epochs)*** |
| 1 | 72 | 39.6 | 40 |
| 2 | 76 | 36.4 | 40 |
| 3 | 81 | 37.3 | 40 |
| 4 | 85 | 43.6 | 40 |

† Table provides statistics for models that predict response to osimertinib. Hardware was a single NVIDIA Tesla V100 32G GDDR6 RAM.

‡ Each QMS feature set is an array of size (633 cell lines x 702 genes), in which each gene is represented by a value describing its mutational state.

* Other columns are: Max Memory is the maximum memory allocated to tensors held on the GPU during training; Train Time is the number of minutes needed to train a model; Early Stop Tolerance (Epochs) is the number of epochs a model can continue to train with no improvement in predictive performance before training is terminated.
