# Supplementary figures and images for: Representing mutations for predicting cancer drug response
Source: Bioinformatics. 2024 Jun 28;40(Suppl 1):i160–8. doi: 10.1093/bioinformatics/btae209 (PMC11256944; doi:10.1093/bioinformatics/btae209)

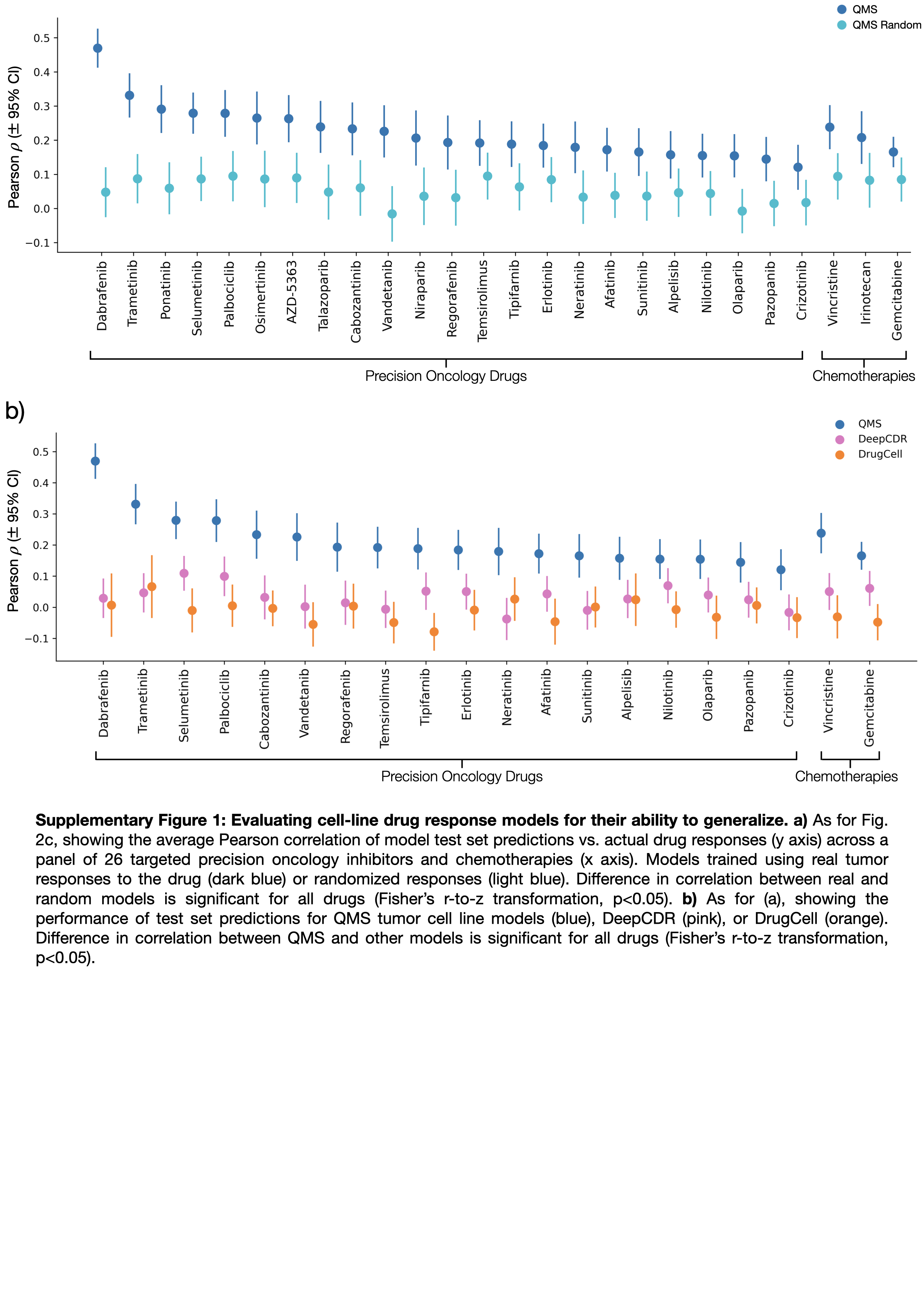

Supplement: btae209_Supplementary_Data [file btae209_supplementary_data.zip › btae209_Supplementary_Data/Wall.178.sup.1.tiff]

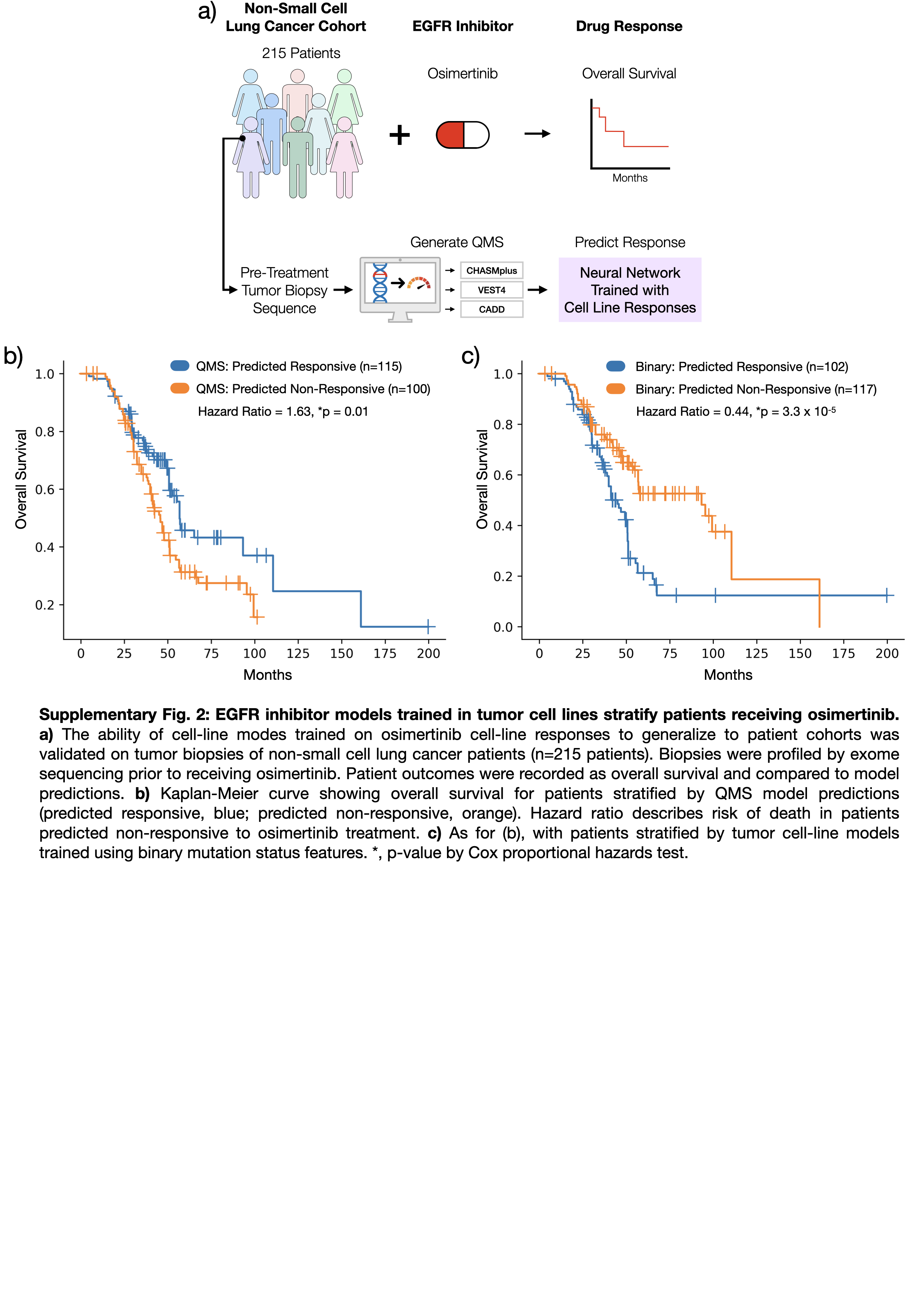

Supplement: btae209_Supplementary_Data [file btae209_supplementary_data.zip › btae209_Supplementary_Data/Wall.178.sup.2.tiff]

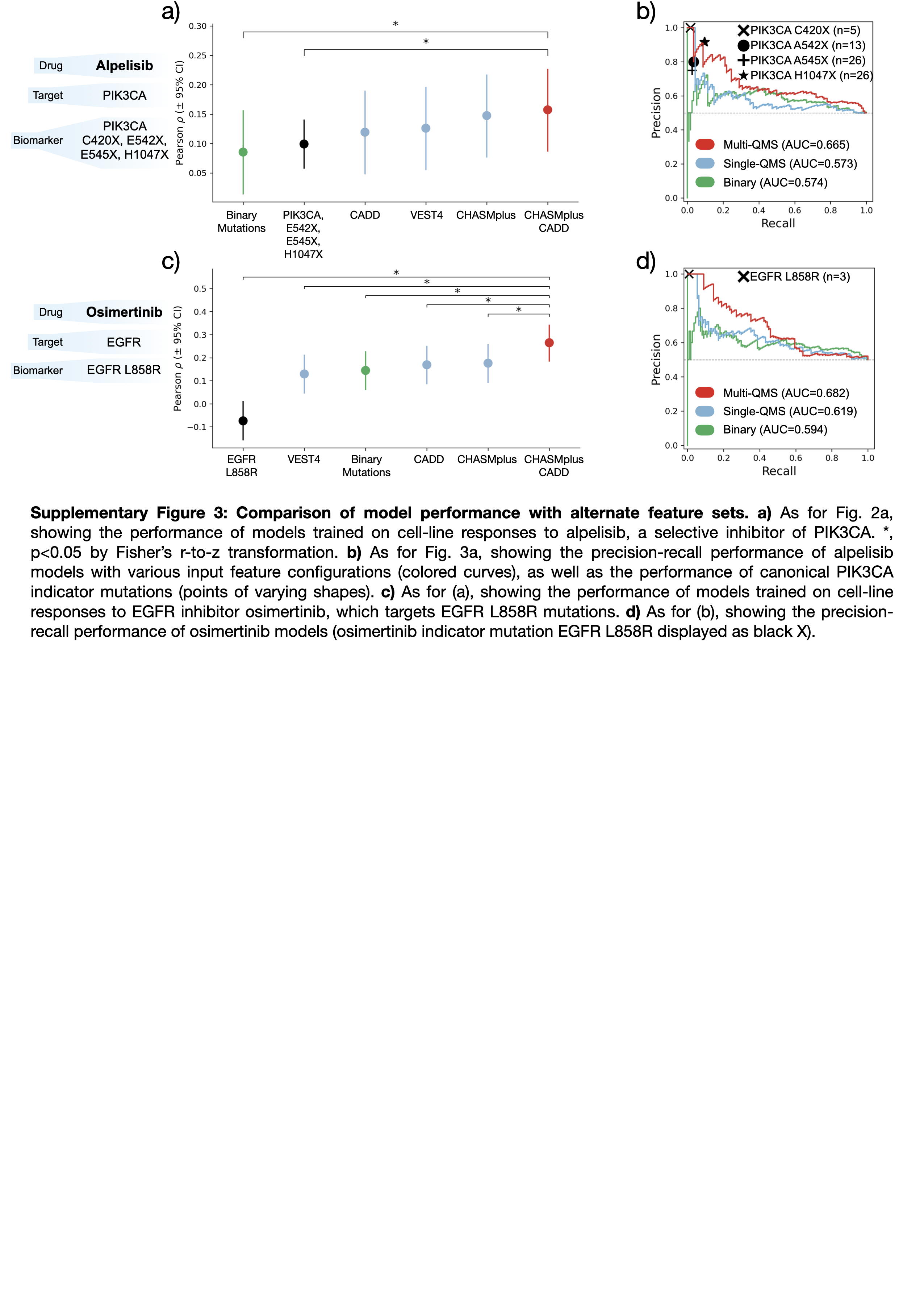

Supplement: btae209_Supplementary_Data [file btae209_supplementary_data.zip › btae209_Supplementary_Data/Wall.178.sup.3.tiff]
